# From assistance to autonomy: AI agent systems in cardiovascular medicine—a review of paradigms, architectures, and clinical translation

**DOI:** 10.3389/fcvm.2026.1838415

**Published:** 2026-08-19

**Authors:** Ye Chen, Xiaoqun Qin, Shouping Chen

**Affiliations:** 1 School of Information Science and Engineering, Hunan International Economics University, Changsha, China; 2 Hunan Nianlun Orthopaedic Hospital Group, Changsha, China

**Keywords:** artificial intelligence, autonomous agents, cardiovascular diseases, clinical governance, digital twins, heart failure, multi-agent systems

## Abstract

**Background:**

Cardiovascular medicine faces persistent implementation gaps driven by workforce shortages, fragmented data systems, and the cognitive burden of complex clinical decision-making—structural constraints that limit the delivery of guideline-directed care. Artificial intelligence (AI) is transitioning from isolated predictive models toward autonomous agent systems capable of perceiving, reasoning, and acting in clinical environments, offering a potential pathway to address these challenges.

**Objective:**

This review synthesizes current evidence on AI agent systems in cardiovascular medicine across four interconnected dimensions: technical paradigms enabling agentic functionality (multi-agent systems, digital twins, multimodal integration), emerging clinical applications across the cardiovascular continuum, and governance frameworks essential for responsible translation.

**Methods:**

We conducted a narrative review of peer-reviewed literature published between January 2020 and March 2026, drawing from PubMed, Web of Science, IEEE Xplore, and Scopus databases. Search terms included combinations of “artificial intelligence”, “AI agents”, “autonomous agents”, “multi-agent systems”, “large language models”, “cardiovascular diseases”, “heart failure”, “digital twins”, and “clinical decision support”. Emphasis was placed on high-quality original research, systematic reviews, and position papers from major cardiovascular societies, with particular attention to developments from 2024 to 2026.

**Results:**

Agentic AI systems should function as augmented intelligence—enhancing rather than replacing clinical judgment—to close implementation gaps in cardiovascular care. Multi-agent architectures, digital cardiovascular twins, and multimodal integration are emerging as core technical paradigms. The ClinNoteAgents system demonstrates high extraction fidelity (conditional accuracy ≥90%) for clinical variables while achieving 60%–90% text reduction. Digital twin applications span therapy planning, risk prediction, and monitoring, with 69% relying on mechanistic models and 76% utilizing imaging data for personalization. Heart failure has emerged as a paradigmatic use case, driven by structural workforce gaps and the ARPA-H ADVOCATE initiative launched in January 2026. The C.A.R.D.I.O. framework (Clinical validation, Auditability, Risk stratification, Data privacy, Integration, Ongoing vigilance) and the CURACO framework (Clinical safety, Understanding, Research-informed care, Authentic patient-centred approaches, Conscientious ethics, Optimised technology) provide governance structures for responsible deployment. A rapid systematic review of 13 studies including 22,641 participants found that 85% of AI interventions improved cardiovascular outcomes, with mortality reductions of 0.8%–12% and major adverse cardiovascular event reductions of 4%–12%.

**Conclusion:**

Cardiovascular medicine stands at an inflection point. The transition from assistance to autonomy requires rigorous fit-for-purpose evaluation, transparent interpretability mechanisms, and robust governance frameworks. Agentic AI systems should function as augmented intelligence—enhancing rather than replacing clinical judgment—to close implementation gaps in cardiovascular care.

## 1 Introduction

Cardiovascular disease remains the leading cause of mortality worldwide, accounting for approximately 18 million deaths annually, with global prevalence continuing to rise across both developed and developing nations (1, 2). Despite decades of progress in preventive strategies, guideline-directed therapies, and interventional techniques, a persistent “implementation gap” separates what is clinically recommended from what is delivered in practice. This gap is particularly evident in preventive cardiology, where fewer than one-quarter of high-risk patients achieve recommended low-density lipoprotein cholesterol targets, and over 40% of patients with established cardiovascular disease have uncontrolled blood pressure despite being on medication (3).

These implementation failures reflect structural constraints: severe time pressure, workforce shortages, fragmented data management, and the cognitive burden of synthesizing complex information from electronic health records during brief consultations. International surveys indicate that less than half of physicians regularly employ validated cardiovascular risk calculators, relying instead on clinical judgment that underestimates risk in nearly two-thirds of high-risk patients (4). The global burden of cardiovascular diseases underscores the critical need for timely, accurate, and efficient diagnostic strategies that can overcome these structural barriers.

Artificial intelligence has been heralded as a solution to these challenges. Over the past decade, predictive AI models have demonstrated remarkable capabilities—from detecting reduced ejection fraction from standard electrocardiograms to identifying coronary artery calcification on chest computed tomography scans obtained for other indications (5). Machine learning and deep learning have significantly advanced areas such as echocardiography, electrocardiography, computed tomography angiography, and predictive analytics (6). AI algorithms have demonstrated superior performance in identifying subtle patterns and anomalies, reducing inter-observer variability, and facilitating earlier detection of cardiovascular conditions (7).

More recently, large language models (LLMs) have captured clinical imagination by passing medical licensing examinations and generating coherent clinical documentation. A systematic review by Patel and colleagues evaluating AI in cardiovascular diagnostics found that AI systems consistently achieve higher diagnostic accuracy, reduce inter-observer variability, and facilitate earlier detection of cardiovascular conditions, leading to improved patient outcomes (8). However, as recent JACC editorials have emphasized, “papers that focus solely on retrospective model performance without a compelling case for clinical relevance, or that fail to articulate a pathway to implementation, will generally not meet the bar” (9).

Despite the burgeoning literature on AI in cardiology, a comprehensive synthesis focusing specifically on the paradigm shift toward autonomous agentic systems, their architectural foundations, and the governance frameworks essential for their clinical translation remains lacking. This review aims to bridge this gap by arguing that cardiovascular medicine is currently undergoing a fundamental paradigm shift—from isolated AI tools toward autonomous agent systems. This shift is not merely technical but conceptual: rather than treating AI as a tool that clinicians query, we are moving toward systems that perceive clinical contexts, reason about complex information, and act—within defined boundaries—to support or execute clinical tasks (10).

### 1.1 The structural imperative: why cardiovascular medicine needs agentic AI

The rationale for agentic AI in cardiology extends beyond technological capability to structural necessity. In January 2026, the Advanced Research Projects Agency for Health (ARPA-H) launched the ADVOCATE (Agentic AI-EnableD CardioVascular CAre TransfOrmation) initiative, representing a watershed moment for autonomous clinical AI (11). The program aims to transform advanced cardiovascular disease management with an agentic AI system that can provide 24/7 holistic clinical care.

The ARPA-H initiative explicitly frames the problem: over 200,000 Americans die each year from preventable impacts of cardiovascular disease, yet nearly half of American counties lack a cardiologist (11). Despite spending almost half a trillion dollars on heart disease alone, the United States experiences much worse outcomes than many other comparable nations. Treatments for managing heart health with diet, exercise, and affordable medications are simple, inexpensive, and effective—yet many patients across the country are missing out on life-saving care and interventions simply because no specialist is available.

The ADVOCATE program's transformative goal is to create a first-of-its-kind, reliable, FDA-authorized clinical agentic AI system that serves around the clock as a new, digital member of the clinical care team (11). This initiative explicitly includes collaboration with the Centers for Medicare and Medicaid Services and the FDA to establish review and reimbursement pathways, signaling that autonomous clinical agents are moving from research concept to implementation focus.

### 1.2 Defining agentic AI in cardiovascular context

To understand this paradigm shift, it is essential to distinguish among three AI paradigms that are often conflated (12):

Predictive AI analyzes existing data to infer or forecast specific outcomes. Examples include models that predict heart failure readmission or detect atrial fibrillation from wearable sensors. These models are deterministic and relatively amenable to validation in traditional clinical trials. However, they typically operate as isolated classifiers rather than integrated systems.

Generative AI creates novel content—text, images, or synthetic data—based on training distributions. LLMs such as GPT-4 exemplify this paradigm, enabling applications from clinical note drafting to patient education. Yet these systems are probabilistic rather than deterministic, producing outputs that may vary across queries and contexts. The risk of “hallucinations”—plausible but factually incorrect content—remains a critical safety concern (13).

Agentic AI represents the frontier: systems capable of perceiving, deciding, and acting autonomously to achieve defined objectives. Unlike predictive or generative models, agents operate within dynamic environments, guided by goals rather than static patterns. They can decompose complex tasks, invoke tools (such as risk calculators or imaging analysis pipelines), synthesize multimodal information, and iterate based on feedback (14). In cardiology, a future agent might simultaneously analyze electrocardiograms, laboratory results, vital signs, medication histories, and clinical notes to synthesize a comprehensive patient overview—a task that currently requires coordination across multiple clinicians.

The structure of this review is as follows: Section 2 examines technical architectures enabling agentic functionality, including multi-agent systems, digital twins, and multimodal integration. Section 3 explores emerging clinical applications across the cardiovascular continuum, with emphasis on heart failure as a paradigmatic use case. Section 4 synthesizes governance frameworks for responsible translation, including the C.A.R.D.I.O. framework, CURACO framework, and Total Product Lifecycle considerations. Section 5 discusses current challenges and future directions, followed by conclusions.

**Figure 1:**
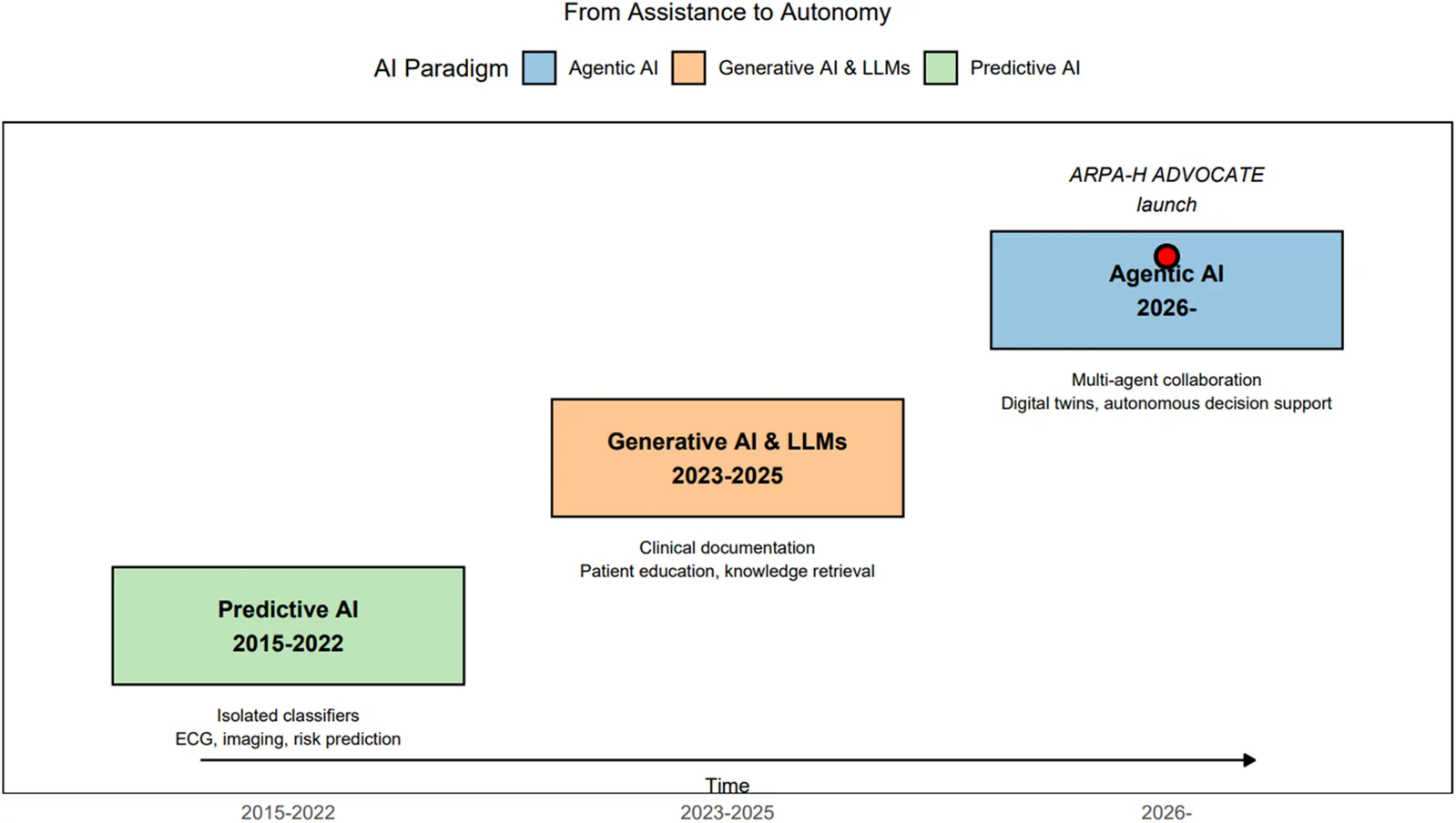
Paradigm shift in cardiovascular AI: from assistance to autonomy. Schematic timeline illustrating the three-stage evolution of artificial intelligence in cardiovascular medicine. Predictive AI (2015–2022): Isolated classifiers for electrocardiography, echocardiography, imaging, and risk prediction, operating as point solutions requiring clinician synthesis. Generative AI and Large Language Models (2023–2025): Probabilistic systems enabling clinical documentation, patient education, and knowledge retrieval, constrained by hallucination risks and lack of deterministic outputs. Agentic AI (2026–): Autonomous systems featuring multi-agent collaboration, digital twin integration, and risk-aligned decision-making. The ARPA-H ADVOCATE initiative (launched January 2026) is positioned as the inflection point marking the transition from assistance to autonomy. Horizontal arrow indicates temporal progression from left to right.

The evolution of artificial intelligence in cardiovascular medicine can be understood as a progressive shift across three distinct paradigms (Figure 1). The first paradigm, predictive AI (2015–2022), was characterized by isolated classifiers applied to specific tasks such as electrocardiogram interpretation, echocardiographic analysis, and risk prediction. These models demonstrated high accuracy but operated as point solutions requiring clinician synthesis of outputs. The second paradigm, generative AI and large language models (2023–2025), introduced capabilities for clinical documentation, patient education, and knowledge retrieval, yet remained constrained by probabilistic outputs and the risk of hallucinations. The current paradigm, agentic AI (2026–), represents a qualitative leap toward systems capable of perceiving multimodal clinical data, reasoning through multi-agent collaboration, and executing risk-aligned actions. The launch of the ARPA-H ADVOCATE initiative in 2026 marks a critical inflection point, signaling the transition from research concepts to implementation-focused autonomous clinical agents. This paradigm shift—from isolated tools to integrated autonomous systems—structures the remainder of this review, with Sections 2–4 examining the architectural foundations, clinical applications, and governance frameworks that define the agentic era.

## 2 Technical architectures: from models to agents

The transition from isolated AI models to autonomous agents requires fundamental shifts in system architecture. This section examines three emergent paradigms: multi-agent collaboration, digital cardiovascular twins, and multimodal integration.

### 2.1 Multi-agent systems: decomposing clinical complexity

Multi-agent systems represent the frontier of AI in cardiovascular medicine. Rather than relying on a single model, these systems employ specialized agents that collaborate to accomplish complex tasks. An agent in this context is defined as a system that can perceive its environment, make decisions, and take actions to achieve specified goals (15).

In cardiovascular applications, multi-agent architectures offer several advantages: decomposition of complex tasks into manageable subtasks, specialization across different clinical domains, verifiability through traceable reasoning paths, and resilience where failure in one agent can be contained (16).

#### 2.1.1 Clinnoteagents: a paradigmatic multi-agent system

A notable recent advance is ClinNoteAgents, an LLM-based multi-agent framework that transforms free-text clinical notes into structured representations of clinical and social risk factors for association analysis and clinician-style abstractions for heart failure 30-day readmission prediction (17). Evaluated on 3,544 notes from 2,065 patients with a readmission rate of 35.16%, ClinNoteAgents demonstrated high extraction fidelity for clinical variables with conditional accuracy exceeding 90% for multiple vital signs (17). Importantly, the system achieved preservation of predictive signal despite 60%–90% text reduction, providing a scalable and interpretable approach to note-based heart failure readmission risk modeling.

The multi-agent architecture enables specialized agents to handle distinct tasks—one agent might extract vital signs, another identify social determinants of health, another synthesize risk factors—while a coordinating agent integrates outputs. This decomposition addresses a key limitation of traditional computational models for heart failure readmission, which often rely on expert-crafted rules, medical thesauri, and ontologies to interpret clinical notes that may contain misspellings, abbreviations, and domain-specific jargon (17).

#### 2.1.2 Multi-agent collaborative reasoning for clinical problem detection

Complementary work by researchers at MIT and Mass General Hospital has developed a collaborative multi-agent system (MAS) that models a clinical consultation team for automated clinical problem detection from SOAP notes (18). The system uses a Manager agent to orchestrate a dynamically assigned team of specialist agents who engage in hierarchical, iterative debate to reach consensus. Evaluated on a curated dataset of 420 MIMIC-III notes, the dynamic multi-agent configuration demonstrated consistently improved performance in identifying congestive heart failure, acute kidney injury, and sepsis compared to single-agent baselines (18). Qualitative analysis of the agent debates reveals that this structure effectively surfaces and weighs conflicting evidence, though it can occasionally be susceptible to groupthink.

This approach models a clinical team's reasoning process, offering a promising path toward more accurate, robust, and interpretable clinical decision support tools. By simulating the diagnostic reasoning process of synthesizing raw data into an assessment, these systems represent a significant advance over single-model approaches that can lack the robustness required for high-stakes clinical tasks (18).

#### 2.1.3 Heartagent: autonomous differential diagnosis

Further advancing the multi-agent paradigm, Zhou and colleagues developed HeartAgent, an autonomous agent system for explainable differential diagnosis in cardiology (19). In evaluations on the MIMIC dataset, HeartAgent achieved Top-3 diagnostic accuracy improvements exceeding 36% compared to traditional methods. When used to assist clinicians, the system improved physician diagnostic accuracy by 26.9% and explanation quality by 22.7% (19). The system's core innovation lies in its verifiable reasoning trajectory—each step of the diagnostic process can be traced back to original literature or clinical guidelines.

#### 2.1.4 Integration frameworks: blockchain, LLMs, and extended reality

Musamih and colleagues proposed a conceptual integration model that synthesizes AI, LLMs, blockchain, and metaverse-based extended reality (XR) into a unified cardiovascular ecosystem (20). This framework, published in Global Cardiology Science and Practice in February 2026, consolidates the current literature on these technologies and evaluates their roles in cardiology. The authors conducted a structured narrative review using PubMed and Scopus to identify peer-reviewed studies published between 2018 and 2025, analyzing 50 studies across the three technology domains.

Key findings from their analysis: LLMs showed promise in documentation, education, and decision support but faced challenges in accuracy, validation, and bias; blockchain applications improved data exchange, consent management, and provenance tracking, though scalability and interoperability remain unresolved; XR technologies enhanced procedural precision, training, and patient engagement but were limited by cost and validation (20). Their conceptual integration model illustrates how these technologies function within a unified cardiovascular ecosystem where blockchain provides the trust layer, LLMs deliver analytical intelligence, and XR enables immersive interaction.

#### 2.1.5 Implications for agentic systems

The multi-agent architectures described above have direct implications for clinical translation. The decomposition of complex clinical tasks into specialized, verifiable components addresses a core requirement for autonomous systems: traceability. When each agent's reasoning can be audited, the system becomes amenable to regulatory oversight and clinical trust. Furthermore, the ability to invoke external tools (risk calculators, imaging pipelines) rather than generating all outputs from a single model reduces hallucination risks—a critical safety consideration for clinical deployment.

### 2.2 Digital cardiovascular twins: bridging simulation and action

Among the most promising applications of agentic architectures is the digital cardiovascular twin—a dynamic, computational representation of an individual's cardiovascular physiology that enables simulation, prediction, and proactive intervention (21). A comprehensive systematic review by Khoshfekr Rudsari and colleagues, published in Frontiers in Digital Health, examines the current state of digital twins in healthcare across different physiological levels—from cellular to whole-body systems (22).

The review highlights significant achievements including population-scale cardiac modeling (3,461 patient cohort), reduced atrial fibrillation recurrence rates through patient-specific cardiac models, improved brain tumor radiotherapy planning, advanced liver regeneration modeling with real-time simulation capabilities, and enhanced glucose management in diabetes (22). The authors detail the methodological foundations supporting different digital twin implementations, including data acquisition strategies, physics-based modeling approaches, statistical learning algorithms, neural network-based control systems, and emerging artificial intelligence techniques.

#### 2.2.1 Systematic review of cardiovascular digital twins

A more focused systematic review by a research team from multiple institutions examined cardiovascular digital twin systems specifically, analyzing 42 original studies from 330 screened records across 5 databases (23). This review addressed 11 research questions spanning modeling technologies, data infrastructure, clinical applications, clinical impact, implementation barriers, and ethical considerations.

Key findings reveal that most digital twins (29/42, 69%) relied on mechanistic models, while hybrid mechanistic–data-driven approaches and purely data-driven designs were less frequent (13/42, 31%) (23). Only 18 studies explicitly described machine learning or AI, most often deep learning, Bayesian methods, or optimization algorithms. Personalization depended primarily on imaging (32/42, 76%) and electrocardiography or other electrical signals (18/42, 43%). Visualization was dominated (41/42, 98%) by static figures and anatomical snapshots (23).

Clinically, digital twins were most commonly applied to therapy planning, risk prediction, and monitoring. Reported benefits focused on improved decision-making and therapy-related impacts, with occasional (8/42, 19%) reports of increased accuracy or faster diagnosis, but there was limited evidence for downstream improvements in patient outcomes (23).

#### 2.2.2 Tasmurzayev's four-layer architecture

Tasmurzayev and colleagues synthesized 183 studies to propose a comprehensive four-layer architecture for digital cardiovascular twins (21). Layer 1: Sensor Data Collection involves continuous streams from wearable devices (electrocardiography, photoplethysmography, mechanocardiography) combined with clinical records, laboratory biomarkers, and genetic information. Layer 2: Hybrid Analytics integrates hemodynamic models with deep learning, graph networks, and transformer architectures, with Bayesian and Kalman filters managing uncertainty. Layer 3: Decision Support employs domain-tuned medical large language models and autonomous agents to generate personalized alerts, treatment recommendations, and clinical insights. Layer 4: Prospective Simulation—the most distinctive capability—enables prospective modeling that simulates interventions (such as pacing strategies or pharmacotherapy adjustments) before clinical application, closing the prediction-intervention loop (21).

What distinguishes digital twins from conventional predictive models is their closed-loop architecture. As Tasmurzayev and colleagues note, this stack “flags silent pathology weeks in advance and steers proactive personalized prevention” (21). The heart failure context is particularly relevant here. Early detection of decompensation—often occurring days before clinical presentation—is a recognized gap in current care. Digital twins that integrate continuous sensor data with physiological modeling could detect subtle changes, simulate intervention effects, and trigger proactive care before hospitalization becomes necessary (24).

#### 2.2.3 Barriers to digital twin implementation

The systematic review identified key barriers to cardiovascular digital twin implementation: strong model assumptions and simplifications; high computational cost; data quality and availability constraints; limited external validation; and challenges in real-time performance, workflow integration, and usability (23). Explicit discussion of ethical, legal, or governance issues was rare (7/42, 17%), highlighting a critical gap in the literature (23).

#### 2.2.4 Implications for agentic systems

Digital twins represent a critical enabler for autonomous clinical agents. The ability to simulate interventions before deployment—the fourth layer of Tasmurzayev's architecture—addresses a fundamental safety requirement: agents should not act without understanding the likely consequences. However, the current predominance of mechanistic models and limited AI integration suggests that most digital twins remain in the “assistance” rather than “autonomy” paradigm. Future agentic systems will require deeper integration of AI-driven decision-making with mechanistic physiological modeling.

### 2.3 Multimodal integration: beyond text and images

Clinical cardiovascular reasoning inherently integrates diverse data modalities: text (clinical notes, guidelines), images (echocardiography, angiography, computed tomography), signals (electrocardiography), structured data (laboratory values), and increasingly genomics. The challenge of multimodal integration has been a persistent barrier to comprehensive AI systems (25).

Recent advances in multimodal artificial intelligence are beginning to address this barrier. A systematic review by Zhang and colleagues outlines five domains where multimodal AI contributes to heart failure care: risk prediction, precision diagnosis, phenotyping, personalized treatment, and prognosis (26). The authors emphasize that “the powerful data-processing and feature-extraction capabilities of AI enable the effective integration of multimodal data, facilitating the elucidation of mechanistic roles of biomarkers and comorbidities, revealing underlying pathological processes, and advancing the development of personalized therapeutic strategies” (26).

For agentic systems, multimodal integration is not merely a technical achievement but a functional necessity—an autonomous agent managing heart failure must simultaneously interpret text notes, review echocardiographic reports, analyze electrocardiographic trends, and synthesize laboratory values. The Taiwan AICoE's intelligent health agent platform exemplifies this architecture, describing agents that “perform real-time sensing, infer patient conditions using multimodal modeling techniques, predict health trajectories, and propose individualized interventions” (14).

#### 2.3.1 Implications for agentic systems

Multimodal integration directly enables the “perception” function of agentic systems. An agent that cannot interpret both structured data and free-text narratives, or cannot synthesize imaging findings with laboratory values, will fail to achieve the clinical awareness required for autonomous operation. Current multimodal systems, while advancing rapidly, still face challenges in handling the full breadth of cardiovascular data modalities with equal proficiency. Future development should prioritize architectures that maintain performance across modalities while providing interpretability mechanisms that allow clinicians to understand how multimodal inputs inform agent decisions.

### 2.4 The agent architecture: perception, reasoning, action

Drawing on emerging frameworks from both clinical and computer science literature, agentic systems in cardiovascular medicine can be characterized by three functional components (14, 27). A summary of the levels of evidence for these and other key agentic systems is provided in Table 1.

**Table 1:** Levels of evidence for Key AI agent systems in cardiovascular medicine.

System/Framework	Level of Evidence	Key Finding
ClinNoteAgents	Retrospective validation (*n* = 2,065 patients)	Conditional accuracy ≥90% for clinical variables
HeartAgent	Proof-of-concept (MIMIC dataset)	36% improvement in Top-3 diagnostic accuracy
Multi-agent collaborative system (MIT/MGH)	Retrospective validation (420 MIMIC notes)	Improved detection of HF, AKI, sepsis vs. single agents
Digital cardiovascular twins	Mixed: 69% mechanistic models, 31% hybrid; most in proof-of-concept to retrospective	Limited prospective outcome data (8/42 reported accuracy gains)
Taiwan AICoE intelligent health agent	Conceptual framework	No clinical validation data reported
TeleAI-CVD	Prospective observational	Feasibility demonstrated in outpatient cardiology
ARPA-H ADVOCATE	Programmatic initiative (ongoing)	No published clinical results yet

Perception involves converting raw data into structured representations of clinical state. This includes not only extracting values from electronic health records but also interpreting free text, analyzing images, and synthesizing continuous monitoring streams. Advanced perception systems maintain uncertainty estimates and confidence intervals, enabling downstream reasoning to account for information quality.

Reasoning encompasses the cognitive processes that transform perceived states into decisions. In multi-agent systems, reasoning may be distributed: a risk assessment agent applies deterministic algorithms (such as SCORE2 or pooled cohort equations), a guideline interpretation agent retrieves relevant recommendations, a planning agent simulates intervention sequences, and a critique agent identifies potential errors or omissions. The reasoning architecture must support transparency—enabling clinicians to trace how conclusions were reached.

Action refers to system outputs that affect clinical care. Actions range from low-risk information provision (generating patient education materials) to moderate-risk decision support (recommending medication adjustments) to high-risk autonomous execution (administering therapies). The appropriate level of autonomy must be calibrated to clinical risk and regulatory context.

The ADVOCATE program envisions clinical AI agents that can be trusted to autonomously adjust changes in appointments, medications, diet, and exercise, supported by a supervisory AI “overseer” to monitor clinical AI agents after they have been deployed in clinical practice to ensure their continued safety and efficacy (11).

**Figure 2:**
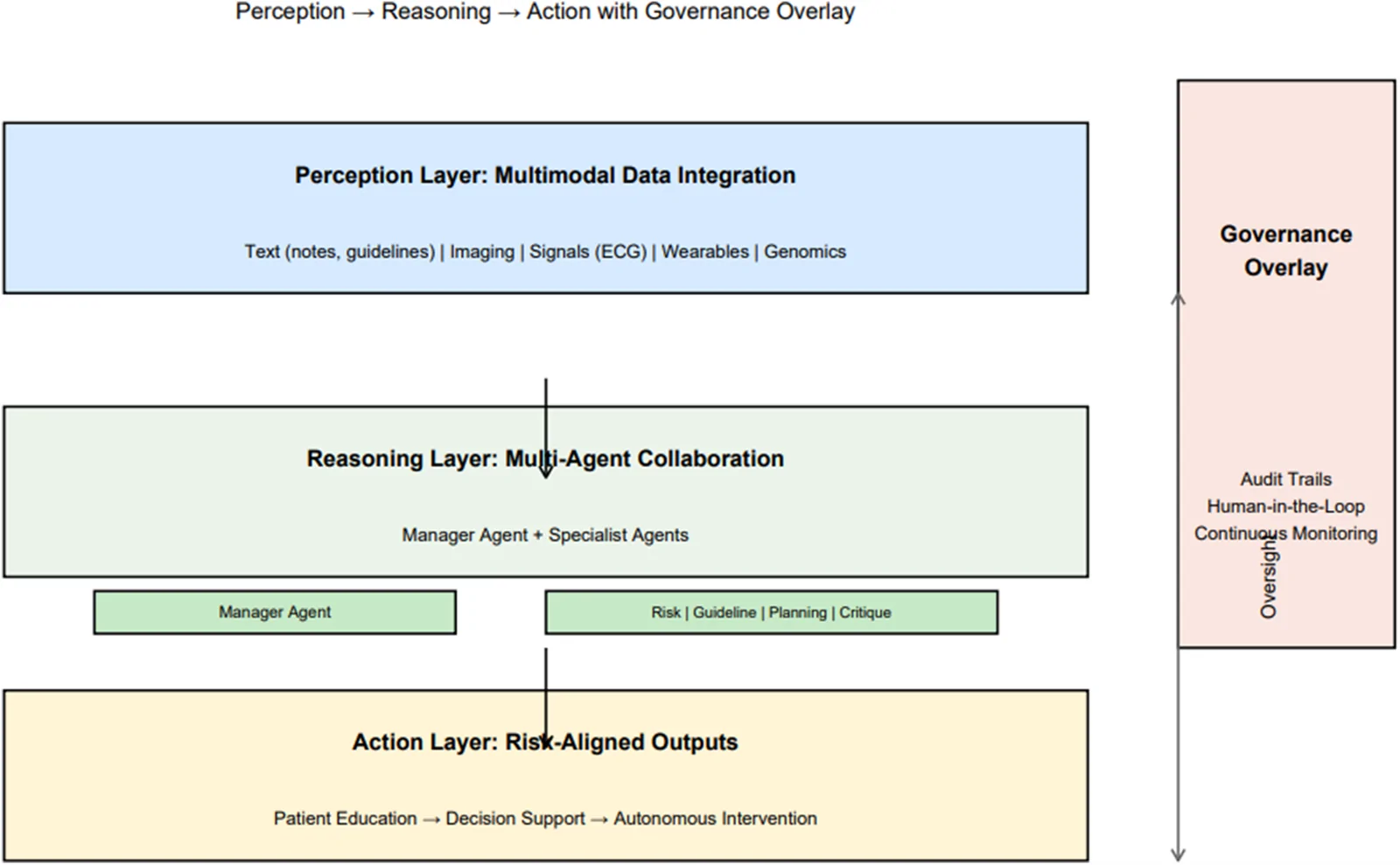
Integrated architecture for cardiovascular AI agents. Three-layer framework defining the functional components of autonomous clinical agents with governance overlay. Perception Layer: Multimodal data integration from electronic health records (text, structured data), imaging (echocardiography, angiography, computed tomography), signals (electrocardiography, wearable sensors), and genomics. Advanced perception systems maintain uncertainty estimates to inform downstream reasoning. Reasoning Layer: Multi-agent collaboration architecture where a Manager agent orchestrates specialized agents—risk assessment, guideline interpretation, treatment planning, and critique—that engage in iterative deliberation to reach consensus. This structure enables verifiable, auditable reasoning trajectories. Action Layer: Risk-aligned outputs calibrated to clinical risk levels—from patient education (low risk) to decision support with clinician approval (moderate risk) to autonomous execution within defined boundaries (high risk). Governance Overlay: Audit trails enabling retrospective review, human-in-the-loop controls maintaining appropriate oversight, and continuous monitoring for performance drift. Arrows indicate bidirectional information flow and feedback loops across layers.

The technical architectures described in this section—multi-agent systems, digital cardiovascular twins, and multimodal integration—can be unified within a three-layer framework that defines the functional components of an autonomous cardiovascular AI agent (Figure 2). At the foundation, the perception layer integrates diverse data modalities: free-text clinical notes, imaging studies (echocardiography, angiography), electrophysiological signals (electrocardiography, wearables), structured laboratory values, and genomic information. Advanced perception systems maintain uncertainty estimates, enabling downstream reasoning to account for information quality. The reasoning layer implements multi-agent collaboration, where a Manager agent orchestrates specialized agents—risk assessment, guideline interpretation, treatment planning, and critique—that engage in iterative deliberation to reach consensus. This architecture, demonstrated in systems such as ClinNoteAgents and HeartAgent, enables verifiable reasoning trajectories that can be audited retrospectively. The action layer calibrates outputs to clinical risk, ranging from low-risk patient education materials to moderate-risk decision support (medication recommendations with clinician approval) to high-risk autonomous execution within defined boundaries. Overlaying these three layers is a governance framework comprising audit trails, human-in-the-loop controls, and continuous performance monitoring—essential mechanisms for maintaining safety and accountability as autonomy increases. This integrated architecture provides the structural foundation for the clinical applications and governance frameworks discussed in Sections 3 and 4.

## 3 Clinical applications across the cardiovascular continuum

### 3.1 Prevention: bridging the implementation gap

Preventive cardiology represents perhaps the most compelling use case for agentic systems. The gap between guideline recommendations and clinical practice is well documented: despite evidence that most cardiovascular events are preventable, less than a quarter of high-risk patients achieve recommended treatment targets (3).

Santos and Dores propose the C.A.R.D.I.O. framework for responsible LLM integration in cardiovascular prevention, synthesizing evidence across three functional domains: patient-facing applications for health literacy and behavior change, clinician-facing applications for decision support and workflow efficiency, and system-facing applications for automated data extraction, registry construction, and quality surveillance (28).

The authors conducted a comprehensive narrative review of literature published between January 2015 and November 2025, concluding that while LLMs generate empathetic, guideline-concordant patient education, they lack the nuance required for unsupervised, personalized advice (28). For clinicians, LLMs effectively summarize clinical notes and draft documentation but remain unreliable for deterministic risk calculations and autonomous decision-making. System-facing applications demonstrate potential for automated phenotyping and multimodal risk prediction (28).

The Belgian Society of Cardiology position paper by Dujardin and colleagues provides a comprehensive roadmap for AI implementation, spanning five phases: development, clinical purpose definition, clinical validation, implementation, and post-market evaluation (4). The authors emphasize that “AI tool development should start from clinical need rather than technical opportunity” and promote “augmented intelligence, designed specifically to enhance human intelligence rather than replace or operate independently from it” (4).

### 3.2 Heart failure: a paradigmatic use case

Heart failure has emerged as the paradigmatic use case for agentic AI—not by accident, but because it embodies the structural, clinical, and technical conditions that make autonomous systems uniquely valuable. Structurally, heart failure affects over 64 million people globally while facing a severe shortage of specialists: nearly half of American counties lack a cardiologist, and the ratio of heart failure specialists to patients continues to narrow. Clinically, heart failure requires continuous monitoring, timely intervention, and coordination across medication optimization, volume management, comorbidity treatment, and patient education—tasks that exceed human capacity at scale. Technically, heart failure generates rich multimodal data (echocardiography, biomarkers, wearable sensors, patient-reported symptoms) that conventional systems struggle to synthesize but agentic architectures are designed to integrate. These converging factors explain why the ARPA-H ADVOCATE initiative selected heart failure as its initial target and why heart failure is rapidly becoming the proving ground for autonomous clinical AI.

#### 3.2.1 The structural imperative for heart failure

Heart failure is emerging as the paradigmatic use case for agentic AI. The reasons are multifaceted: over 64 million people live with heart failure globally (1); management requires coordination across medication optimization, volume management, comorbidity treatment, and patient education; there is a shortage of heart failure specialists relative to demand (11); heart failure generates rich multimodal data that conventional systems struggle to synthesize; and decompensation often develops over days, providing a window for early intervention that current systems frequently miss (26).

The ARPA-H ADVOCATE initiative explicitly focuses on heart failure as the initial target, recognizing that treatments for managing heart health with diet, exercise, and affordable medications are simple, inexpensive, and effective—yet many patients lack access to cardiologists who can oversee this care (11). Krumholz's editorial accompanying the ADVOCATE announcement emphasizes that “heart failure management is an ideal use case for autonomous clinical agents: the condition requires continuous monitoring, timely intervention, and coordination across settings—tasks that exceed human capacity at scale but align with the capabilities of well-designed agent systems” (29).

#### 3.2.2 Clinnoteagents for heart failure readmission

ClinNoteAgents demonstrates the potential of multi-agent systems specifically for heart failure management. By transforming free-text clinical notes into structured representations of clinical and social risk factors, the system enables prediction of 30-day readmission—a key quality metric and target for intervention (17). The system's ability to preserve predictive signal despite 60%–90% text reduction is particularly valuable for healthcare systems with limited structured data infrastructure, offering a scalable and interpretable approach to note-based risk modeling.

#### 3.2.3 Zhang's multimodal framework for heart failure

Zhang and colleagues' systematic review provides a comprehensive overview of AI applications across the heart failure care continuum (26). Their framework encompasses five domains:

Risk prediction: Traditional risk scores for heart failure (such as the Seattle Heart Failure Model) incorporate limited variables and are static. AI approaches enable dynamic risk assessment incorporating longitudinal data. Machine learning models, particularly gradient boosting and deep learning architectures, have demonstrated improved prediction of mortality and readmission compared to conventional approaches (26).

Precision diagnosis: Heart failure diagnosis, particularly the distinction between reduced and preserved ejection fraction, remains challenging. AI-enhanced electrocardiogram analysis has demonstrated accuracy rivaling echocardiography for left ventricular dysfunction detection, enabling opportunistic screening. Ensemble learning approaches combining convolutional neural networks with clinical variables have shown robust performance for early detection of heart failure with preserved ejection fraction (26).

Phenotyping: Heart failure is characterized by substantial heterogeneity. Unsupervised learning approaches have identified clinically meaningful subgroups that may respond differentially to therapies. Using clustering methods on EHR data, Banerjee and colleagues identified five heart failure subtypes—early onset, late onset, atrial fibrillation related, metabolic, and cardiometabolic—with distinct clinical trajectories (30).

Personalized treatment: AI models can simulate treatment effects across patient characteristics, supporting individualized decision-making. This is particularly valuable for heart failure, where medication optimization requires balancing efficacy with tolerability and where patient responses vary substantially (26).

Prognosis: Longitudinal AI models incorporating temporal data have demonstrated improved prediction of clinical outcomes, enabling more accurate prognostic communication and treatment planning (26).

#### 3.2.4 Clinical effectiveness evidence

A rapid systematic review by Adu and colleagues presented at the 2025 American Heart Association Scientific Sessions provides the most comprehensive evidence to date on AI intervention effectiveness in cardiovascular outcomes (31). The review included 13 studies (4 randomized controlled trials, 3 cluster RCTs, 6 observational studies) with 22,641 total participants. Key findings: 85% of studies reported AI interventions improved cardiovascular outcomes; mortality reductions ranged from 0.8% to 12%; heart failure and sepsis mortality odds ratios were 0.39–0.56; major adverse cardiovascular events decreased by 4%–12%; blood pressure reductions ranged from 2.3 to 10.1 mmHg; door-to-treatment time decreased by 86.7 min; medication adherence improved by 40.5%; and LDL cholesterol control improved by 8.7% (31). The authors concluded that AI-guided interventions consistently improve cardiovascular outcomes, with machine learning algorithms and clinical decision support systems demonstrating the strongest evidence.

### 3.3 Diagnostic applications: from images to integrated interpretation

Diagnostic applications of AI in cardiology are among the most mature. Patel and colleagues' systematic review found that machine learning and deep learning have demonstrated significant advancements in areas such as echocardiography, electrocardiography, computed tomography angiography, and predictive analytics (8). AI algorithms have demonstrated superior performance in identifying subtle patterns and anomalies, outperforming conventional methods in several studies.

AI applications in echocardiography have shown enhanced diagnostic accuracy, with algorithms capable of analyzing vast amounts of imaging data rapidly and with high precision, identifying subtle patterns that may be overlooked by human observers (27). Ouyang and colleagues developed a video-based AI model that can assess cardiac function on a beat-to-beat basis from echocardiographic videos, achieving left ventricular ejection fraction measurements approaching human expert accuracy (7).

Similarly, AI-enhanced electrocardiogram analysis has demonstrated accuracy rivaling echocardiography for left ventricular dysfunction detection, enabling opportunistic screening (5). Dhingra and colleagues evaluated AI-ECG performance across diverse image formats, finding that models maintain high accuracy across varied inputs, supporting clinical deployment (5). A comprehensive review by Siontis and colleagues in Nature Reviews Cardiology details the expanding applications of AI-enhanced electrocardiography across arrhythmia detection, cardiomyopathy screening, and valvular heart disease identification (32).

What agentic systems add to diagnostic applications is integration. Current AI diagnostic tools are typically point solutions—a model for electrocardiogram interpretation, a separate model for echocardiography. A clinician must still synthesize these outputs. An agent system could coordinate multiple diagnostic agents, synthesize findings, generate differential diagnoses, and suggest confirmatory tests—approximating the integrated reasoning of a clinical cardiologist (19).

### 3.4 Training and education applications

Beyond direct clinical care, AI agent systems are finding applications in cardiology training and education. Sooltan and colleagues evaluated AI-generated acute coronary syndrome (ACS) management checklists enhanced through the CURACO framework (Clinical safety, Understanding, Research-informed care, Authentic patient-centred approaches, Conscientious ethics, Optimised technology) (33).

In a pilot study of resident doctors rotating through cardiology, the CURACO-enhanced approach demonstrated significant improvements across all domains (*p* < 0.001). ECG interpretation confidence improved dramatically, with 66.7% reporting maximum confidence post-implementation vs. 6.7% pre-implementation. Risk stratification confidence for invasive management increased from 0% to 93.3% for high confidence scores (33). Importantly, 100% of users agreed the checklist helped standardize their approach while maintaining patient-centred focus, and patient understanding components were valued by 80% of users, creating a more holistic approach than traditional implementations (33).

The CURACO framework provides a foundation for developing clinical tools serving both clinical excellence and patient-centred care, demonstrating that AI-derived interventions can be transformed through systematic integration of safety, understanding, research, authenticity, ethics, and technology (33).

A scoping review by Zheng and colleagues examined generative AI in cardiovascular specialty care, analyzing 19 studies that included 9 text generation models, 3 multimodal generation models, 4 temporal prediction models, and 3 chatbots (34). Application scenarios primarily included clinical decision support, patient health management, and nursing education and consultation. The review found that generative AI significantly reduced nursing workload and enabled precise intervention, though limitations in health education and consultation included logical disconnects, poor information quality, and lack of humanistic care (34).

### 3.5 Interventional applications and device management

AI applications are extending into cardiovascular interventions and implantable device management. Qi and colleagues' review in IEEE Reviews in Biomedical Engineering traces the technological evolution of vascular interventional embodied intelligence across perception, planning, navigation, and execution phases (35). The authors propose future directions integrating vascular interventional robotics with AI to enable autonomous or semi-autonomous procedures.

Toth and colleagues' TeleAI-CVD study reported on the integration of telemedicine and AI in outpatient cardiology care, demonstrating the potential of AI-enhanced remote monitoring in cardiovascular disease management (36). Siontis and colleagues’ comprehensive review details AI-enhanced electrocardiography applications in device management, including arrhythmia detection, cardiac resynchronization therapy optimization, and remote monitoring (32).

Krittanawong and colleagues' review in the Journal of the American College of Cardiology systematically examines deep learning applications across cardiovascular medicine, covering image analysis, electrocardiogram interpretation, risk prediction, and drug discovery (6). The authors highlight the transformative potential of deep learning while emphasizing the need for rigorous prospective validation and careful attention to model generalizability.

### 3.6 Longitudinal management: the continuous care paradigm

Perhaps the most distinctive contribution of agentic systems is enabling continuous, rather than episodic, care. Traditional cardiovascular care is episodic—visits every few months, hospitalizations when problems become acute. Agent systems that continuously monitor patients (via wearables, patient-reported data, electronic health record updates) could maintain continuous awareness of clinical status, detecting deterioration early and enabling proactive intervention (14).

The Taiwan AICoE's intelligent health agent platform embodies this continuous care paradigm, describing agents that “perform real-time sensing, infer patient conditions using multimodal modeling techniques, predict health trajectories, and propose individualized interventions” with a full “sense → infer → predict → act → learn” cycle (14).

The ADVOCATE program's vision of a clinical AI agent that serves around the clock as a new, digital member of the clinical care team embodies this continuous care paradigm (11). The program will partner with health systems to successfully deploy the technology across diverse care environments—from major health systems to rural clinics and community settings—with the goal of building trust through rigorous testing and demonstrating measurable improvements in safety, outcomes, and equity (11).

## 4 Governance frameworks for responsible translation

The transition from retrospective model performance to real-world deployment requires rigorous governance frameworks. This section synthesizes emerging guidance from cardiovascular societies, regulatory bodies, and research consortia on the standards needed for responsible AI implementation.

### 4.1 The total product lifecycle framework for generative AI

Armoundas and Singh propose a GenAI-adapted Total Product Lifecycle (TPLC) framework for developers, healthcare providers, and clinicians, published in European Heart Journal—Digital Health (37). The authors emphasize that generative AI-enabled medical devices require adapting the established TPLC paradigm to non-deterministic, rapidly evolving software.

The framework converts TPLC principles into an operational checklist covering: data governance and distribution-aware performance claims in pre-market evaluation; predetermined change control plans (PCCP) based updates with predefined acceptance criteria; and auditable logging, human-in-the-loop controls, and drift surveillance in post-market monitoring (37).

#### 4.1.1 Pre-market evaluation considerations

The authors identify key challenges for pre-market evaluation of generative AI-enabled devices. Performance metrics typically vary with device intended use and are modality-specific. Since generative AI-enabled devices do not act on a human but on data that came from a device attached to a human, the data are continuously changing—performance cannot be assessed on prior data, as drift will make impossible the “equivalence” of a generative AI device with another device at a prior time point (37).

#### 4.1.2 Post-market monitoring

Post-market strategy monitoring starts with pre-market design. The complex landscape in which generative AI models are deployed in healthcare environments underscores the emphasis needed on determining and establishing monitoring capabilities, methodologies, and metrics to effectively monitor and evaluate post-market performance through risk management strategies, user training, accountability, and continuous monitoring (37).

Key challenges in evaluating safety and effectiveness include continuous change resulting from live localized data, user interactions, and changing conditions (such as changes in technologies acquiring the data, patient demographics). There is an increased need for transparency and explainability with respect to training data, as well as performance variability evaluation during post-market monitoring—for example, by employing confidence intervals to demonstrate reliability of outputs, whereas high variability should increase post-market monitoring (37). A human expert in the loop is necessary to evaluate safety, effectiveness, and performance variability.

### 4.2 The FDA framework: labeling for transparency and trust

The U.S. Food and Drug Administration has actively engaged in developing frameworks for AI-enabled medical devices. In January 2026, the FDA convened a CERSI webcast on “Labeling for Transparency and Trust in AI/ML-enabled Cardiac Devices and Software: Patient and Clinician Needs” (38). Research presented at this meeting demonstrated that communicating key elements—such as regulatory approval, device performance, provider oversight, and added value of AI—significantly increased patient trust (14%–19%) and willingness to use (13%–18%) AI-enabled cardiac devices (38).

The meeting identified 12 core communication elements needed by patients and developed conceptual label prototypes and an evidence-based informed labeling framework to support human-centered adoption of AI in medicine (38). This work represents an important step toward standardizing how AI capabilities and limitations are communicated to end-users.

### 4.3 The C.A.R.D.I.O. framework

The C.A.R.D.I.O. framework is summarised in Table 2. Building on the TPLC approach, Santos and Dores propose the C.A.R.D.I.O. framework for responsible LLM integration in cardiovascular prevention, with relevance extending to agentic systems (28):

**Table 2:** The C.A.R.D.I.O. framework for responsible LLM integration in cardiovascular prevention.

Principle	Definition	Clinical translation
Clinical validation	LLMs should be deployed only as supervised “reasoning engines” that augment, rather than replace, clinician judgment. Validation must assess both technical performance and clinical utility.	Requires prospective studies demonstrating improved outcomes, not just technical accuracy.
Auditability	The ability to trace system outputs to inputs and reasoning steps is essential for accountability. Agentic systems should maintain comprehensive logs enabling retrospective audit.	Enables root cause analysis when errors occur; supports regulatory review.
Risk stratification	AI systems should incorporate explicit risk awareness, adjusting behavior based on clinical risk level.	Low-risk applications (patient education) may permit greater autonomy than high-risk applications (medication adjustment).
Data privacy	Compliance with data protection regulations (GDPR, HIPAA) and implementation of privacy-preserving techniques (differential privacy, federated learning) are essential.	Requires architecture that minimizes data exposure while maintaining performance.
Integration	AI systems must integrate with existing clinical workflows rather than requiring clinician adaptation. Integration planning should be explicit in system development.	Addresses a primary barrier to adoption—workflow disruption.
Ongoing vigilance	Continuous monitoring post-deployment is essential, with clear protocols for performance degradation detection and system modification or withdrawal when indicated.	Implements the TPLC principle of lifecycle management.

The authors emphasize that “LLMs could help mitigate structural barriers in CV prevention but should presently be deployed only as supervised ‘reasoning engines’ that augment, rather than replace, clinician judgment” (28).

### 4.4 The CURACO framework

The CURACO framework is summarised in Table 3. The CURACO framework, developed for AI-derived clinical tools, emphasizes six principles (33):

**Table 3:** The CURACO framework for AI-derived clinical tools.

Principle	Definition	Clinical translation
Clinical safety	Safety-first protocols are embedded throughout design and deployment.	Requires explicit risk assessment and mitigation strategies.
Understanding	Ensuring users understand tool capabilities and limitations.	Supports informed use and prevents over-reliance.
Research-informed care	Evidence-based medicine foundation for all recommendations.	Requires grounding in clinical guidelines and validated evidence.
Authentic patient-centred approaches	Maintaining patient-centredness throughout care.	Ensures technology serves patient needs rather than imposing technical constraints.
Conscientious ethics	Considering ethical implications across the lifecycle.	Addresses bias, equity, and accountability.
Optimised technology	Technical optimization and integration for real-world use.	Balances performance with usability and reliability.

This framework emerged from the pilot evaluation of AI-generated ACS checklists, demonstrating that AI-derived interventions can be transformed through systematic integration of these principles. The framework provides foundation for developing clinical tools serving both clinical excellence and patient-centred care (33).

### 4.5 The JACC stage-based evaluation framework

JACC editors have articulated expectations for AI research across development stages (9) (Table 4):

**Table 4:** The JACC stage-based evaluation framework for AI research in cardiovascular care.

Stage	Focus	Key activities
Stage 1	Initial development and validation	Understanding clinical need, model development, validation, performance against benchmarks
Stage 2	Implementation in real-world settings	Workflow integration, feasibility assessment, preliminary impact evaluation
Stage 3	Broader deployment and evaluation	Clinical impact assessment, value creation in healthcare delivery
Stage 4	Long-term monitoring or iterative updates	Performance change over time, continual learning, adaptive updates

The editors note that “although all stages may yield JACC-worthy manuscripts, those including later stages, particularly stages 2–4, which describe real-world implementation and impact, will generally be prioritized” (9).

### 4.6 International regulatory landscape

The regulatory landscape for AI in healthcare is evolving rapidly across jurisdictions. In the United States, the FDA has cleared over 600 AI algorithms, with approximately 10% focused on cardiovascular care, primarily in radiology and implantable devices (39). The FDA's cross-center collaboration framework outlines how the Center for Biologics Evaluation and Research, Center for Drug Evaluation and Research, Center for Devices and Radiological Health, and Office of Combination Products coordinate on AI medical product regulation (39).

In Europe, the EU AI Act will take full effect in August 2026, establishing a risk-based framework for AI systems (40). High-risk AI systems (which likely include most clinical agentic systems) must meet requirements for risk management, data governance, transparency, human oversight, accuracy, and robustness. Conformity assessment requires involvement of notified bodies, with additional requirements for systems used in healthcare (40).

The World Health Organization has issued guidance on the ethics and governance of AI for health, emphasizing that AI should promote health equity, protect privacy, ensure transparency, maintain human autonomy, and establish accountability mechanisms (41).

### 4.7 Ethical considerations and human-centered AI

Beyond technical governance frameworks, broader ethical considerations must guide agentic AI development. Barry and colleagues in JAMA propose a human-centered AI framework emphasizing transparency and trust (42). Their research demonstrates that communicating key elements such as regulatory approval, device performance, provider oversight, and added value of AI significantly increases patient trust and willingness to use AI-enabled devices (42).

Reddy and colleagues propose a four-layer governance model for healthcare AI: data layer (data quality, privacy, security), algorithm layer (validation, interpretability, bias), clinical layer (clinical effectiveness, workflow integration), and societal layer (ethics, equity, accountability) (43).

Kelly and colleagues analyze key challenges for delivering clinical impact with AI, including evidence generation, clinical integration, human-computer interaction, regulatory approval, reimbursement models, and continuous monitoring (44). Wiens and colleagues propose a roadmap for responsible machine learning in healthcare covering fairness, interpretability, privacy, robustness, reproducibility, transparency, clinical validation, and continuous monitoring (45).

Challen and colleagues analyze bias and clinical safety concerns in AI medical applications, proposing methodological frameworks for bias detection and mitigation (46). Topol discusses the implications of multimodal AI for medical practice, highlighting challenges in algorithm transparency, data privacy, and clinical responsibility (47).

Char and colleagues address ethical challenges in implementing machine learning in healthcare, including informed consent, transparency, clinical responsibility, algorithm bias, and fairness (48). McCradden and colleagues critically analyze the ethical limitations of algorithmic fairness solutions, noting that technical approaches alone cannot address structural healthcare inequities (49). Jobin and colleagues map the global landscape of AI ethics guidelines, identifying core principles including transparency, justice and fairness, non-maleficence, responsibility, privacy, and interpretability (50).

Meskó and Topol emphasize the imperative for regulatory oversight of LLMs in healthcare, calling for dynamic regulatory frameworks that can adapt to the continuous learning characteristics of these systems (51).

## 5 Discussion: challenges and future directions

### 5.1 Summary of key findings

This review has synthesized current evidence on AI agent systems in cardiovascular medicine across three domains:

Technical architectures are evolving from single models to multi-agent systems, digital twins, and multimodal integration. ClinNoteAgents demonstrates high extraction fidelity (≥90% conditional accuracy) for clinical variables while achieving 60%–90% text reduction (17). Digital twin applications span therapy planning, risk prediction, and monitoring, with 69% relying on mechanistic models and 76% utilizing imaging data for personalization (23).

Clinical applications are emerging across the cardiovascular continuum, with heart failure as a paradigmatic use case driven by structural workforce gaps and supported by the ARPA-H ADVOCATE initiative launched in January 2026 (11). Clinical effectiveness evidence from 13 studies including 22,641 participants demonstrates that 85% of AI interventions improve cardiovascular outcomes, with mortality reductions of 0.8%–12% and MACE reductions of 4%–12% (31).

Governance frameworks—including the TPLC framework for generative AI (37), C.A.R.D.I.O. framework (28), CURACO framework (33), and JACC stage-based evaluation framework (9)—provide essential guidance for responsible translation from research to practice. FDA initiatives on labeling for transparency (38) and international regulatory frameworks (40, 41) are establishing the infrastructure for clinical deployment.

### 5.2 Current challenges

#### 5.2.1 Technical challenges

Hallucinations and reliability concerns remain significant barriers for generative AI in clinical contexts. Armoundas and Singh emphasize that the stochastic sampling and non-deterministic nature of generative AI requires safe use to hinge on robust risk management strategies (37). The complexity of true multimodal integration, interpretability of deep learning systems, and data privacy requirements for agentic systems that require extensive patient data access all pose ongoing challenges (25, 26).

The digital twin systematic review identified strong model assumptions and simplifications, high computational cost, data quality and availability constraints, limited external validation, and challenges in real-time performance as key technical barriers (23).

#### 5.2.2 Clinical challenges

The field lacks consensus validation standards for agentic AI. While systematic reviews demonstrate that AI systems achieve higher diagnostic accuracy and reduce inter-observer variability (8), most evidence comes from retrospective studies with limited prospective validation. The digital twin systematic review found limited evidence for downstream improvements in patient outcomes, with only 8 of 42 studies (19%) reporting increased accuracy or faster diagnosis (23).

Workflow integration remains challenging, and clinician training for AI-augmented practice is inadequate (4). Kelly and colleagues identify clinical integration as a key challenge for delivering clinical impact with AI, noting that successful implementation requires understanding existing workflows, identifying integration points, and designing systems that complement rather than disrupt clinical practice (44).

#### 5.2.3 Regulatory challenges

Regulatory frameworks for AI—particularly agentic AI—are still evolving. The EU AI Act's requirements for high-risk systems will take full effect in August 2026, with implications for cardiovascular AI applications (40). In the United States, FDA guidance for AI as a medical device continues to develop (39). The ARPA-H ADVOCATE initiative's engagement with CMS and FDA suggests progress, but sustainable reimbursement models remain under development (11).

#### 5.2.4 Implementation barriers

The digital twin systematic review identified key implementation barriers: data integration challenges, computational scalability limitations, digital equity concerns, and validation framework gaps (23). Explicit discussion of ethical, legal, or governance issues was rare (7/42, 17%), highlighting a critical gap in the literature (23).

Challen and colleagues emphasize that addressing bias and ensuring clinical safety requires systematic approaches to bias detection and mitigation throughout the AI lifecycle (46). McCradden and colleagues caution that technical approaches to algorithmic fairness alone cannot address structural healthcare inequities, calling for broader systems-level interventions (49).

### 5.3 Future research priorities

Based on the evidence synthesized in this review, we identify six priority areas for future research aligned with the development goals outlined in the literature:

#### 5.3.1 Priority 1: prospective clinical trials

The field requires prospective trials evaluating agentic AI systems in real-world clinical settings, assessing not only technical performance but clinical outcomes, workflow impact, and user experience (9). The ADVOCATE program's goal of developing a reliable, FDA-authorized clinical agentic AI system will require rigorous clinical validation across diverse care environments (11). Jain and colleagues' stage-based framework provides a roadmap for progressing from retrospective validation through real-world implementation to long-term monitoring (9).

#### 5.3.2 Priority 2: integration of digital twins with multi-agent systems

Current digital twins predominantly rely on mechanistic models (69%) with limited AI integration (31%) (23). Future research should focus on developing fourth-generation digital cardiovascular twins that fully integrate mechanistic modeling with data-driven AI approaches (21). The Tasmurzayev four-layer architecture provides a framework for achieving “perception → simulation → decision → action” closed-loop systems (21). Embedding multi-agent systems within digital twins could enable autonomous intervention planning while maintaining human oversight.

#### 5.3.3 Priority 3: human-AI collaboration optimization

Optimizing the interface between human clinicians and AI agents requires systematic study (4, 44). The multi-agent systems that model clinical consultation teams (18) and the CURACO framework's emphasis on patient-centred approaches (33) offer starting points. Future research should identify tasks best performed by AI vs. those requiring human judgment, and develop effective collaboration mechanisms that enhance rather than replace clinical expertise.

#### 5.3.4 Priority 4: standardized governance and regulatory pathways

The field requires standardized governance frameworks for agentic AI. The TPLC framework for generative AI (37), C.A.R.D.I.O. framework (28), and CURACO framework (33) provide foundational elements that should be integrated into formal regulatory guidance. Future work should focus on operationalizing these frameworks into concrete regulatory pathways, building on FDA's labeling transparency research (38) and EU AI Act requirements (40).

#### 5.3.5 Priority 5: open-source model development and validation

Tarabanis and colleagues' evaluation demonstrating that open-source models like DeepSeek R1 can achieve superior performance (86.9% accuracy) compared to proprietary models on cardiology knowledge assessment (52) suggests that open-source approaches merit further investment. Future research should develop cardiology-specialized open-source models with local deployment capabilities, reducing data privacy concerns while maintaining transparency and configurability. Standardized validation protocols for open-source medical AI should be established.

#### 5.3.6 Priority 6: equity and fairness assessment

Ensuring equitable performance across populations—including race, ethnicity, socioeconomic status, and geographic location—requires systematic evaluation (48, 49). The ADVOCATE program explicitly aims to demonstrate measurable improvements in equity alongside safety and outcomes (11). Future research should develop methods for detecting and mitigating bias across the AI lifecycle, with particular attention to populations underrepresented in training data.

### 5.4 Toward trustworthy autonomy

The transition from assistance to autonomy in cardiovascular AI is underway, but its trajectory will be shaped by collective choices about system design, evaluation, and governance. Drawing on the frameworks reviewed here, several principles can guide this transition:

Augmentation, not replacement: Agentic AI systems should augment clinical judgment rather than replace it. As the Belgian authors emphasize, the goal is “augmented intelligence, designed specifically to enhance human intelligence rather than replace or operate independently from it” (4). This principle aligns with the C.A.R.D.I.O. framework's emphasis on clinical validation as supervised “reasoning engines.”

Risk-aligned autonomy: The level of system autonomy should align with clinical risk. Low-risk applications (patient education) may permit greater autonomy, while high-risk applications (medication adjustment) should maintain human oversight and final decision authority (28). This directly operationalizes the C.A.R.D.I.O. principle of risk stratification.

Transparency by design: Agentic systems should be designed to enable understanding of their reasoning, with mechanisms for retrospective audit and explanation (37, 42). This implements the C.A.R.D.I.O. principle of auditability and the CURACO principle of understanding. FDA's labeling research demonstrates that transparent communication of key elements significantly increases patient trust and willingness to use AI-enabled devices (38).

Continuous evaluation: AI systems are not static; their performance evolves with clinical practice, patient populations, and underlying data distributions (37). This aligns with the C.A.R.D.I.O. principle of ongoing vigilance and the TPLC framework's emphasis on post-market monitoring. The JACC stage-based framework provides a structure for progressing evaluation across the lifecycle.

Stakeholder engagement: Clinicians, patients, health systems, and regulators should be engaged throughout the development and deployment lifecycle, ensuring that systems address real needs and are acceptable to those who use and are affected by them (4, 43). This reflects the CURACO principle of authentic patient-centred approaches and the C.A.R.D.I.O. principle of integration.

The ARPA-H ADVOCATE initiative's focus on heart failure management suggests that autonomous clinical agents are moving from research concept to implementation focus (11). With appropriate governance frameworks and rigorous evaluation, these systems may help address the structural workforce gaps that limit cardiovascular care delivery. However, achieving this potential requires sustained commitment to responsible innovation—balancing technical ambition with careful attention to safety, equity, and human values.

## 6 Conclusion

Cardiovascular medicine stands at an inflection point. The convergence of predictive AI, generative AI, and agentic architectures is enabling a fundamental shift—from isolated tools that clinicians query toward autonomous systems that perceive, reason, and act in clinical environments. This transition from assistance to autonomy is driven not merely by technical capability but by structural necessity: workforce shortages, fragmented data systems, and implementation gaps that limit cardiovascular care delivery (4, 11).

The evidence synthesized in this review demonstrates that agentic AI systems are emerging across the cardiovascular continuum. Multi-agent architectures such as ClinNoteAgents demonstrate high extraction fidelity for clinical variables while achieving substantial text reduction (17). Digital twin applications span therapy planning, risk prediction, and monitoring, with substantial potential to advance precision cardiology (21, 23). The ARPA-H ADVOCATE initiative represents a watershed moment, signaling that autonomous clinical agents are moving from research concept to implementation focus (11).

Clinical effectiveness evidence is accumulating, with a rapid systematic review of 13 studies including 22,641 participants demonstrating that 85% of AI interventions improve cardiovascular outcomes, with clinically meaningful reductions in mortality, MACE, blood pressure, and door-to-treatment time (31). This evidence base, while promising, requires further strengthening through prospective randomized controlled trials that assess not only technical performance but clinical outcomes, workflow impact, and user experience (9).

However, the path to clinical integration requires rigorous attention to evaluation, governance, and oversight. The TPLC framework for generative AI (37), C.A.R.D.I.O. framework (28), CURACO framework (33), JACC stage-based evaluation framework (9), and FDA labeling transparency initiatives (38) provide essential guidance. The shift to autonomy does not imply the replacement of clinical judgment; rather, the goal should be augmented intelligence—systems that enhance human capability while maintaining clinician authority and accountability (4).

The coming years will be critical in determining whether agentic AI systems fulfill their potential. Success will require sustained collaboration across disciplines—clinicians, data scientists, regulators, health systems, and patients—working together to develop, evaluate, and deploy systems that are safe, effective, and aligned with human values. By embracing rigorous evaluation, responsible governance, and human-centered design, the cardiovascular community can help ensure that agentic AI systems deliver on their promise—augmenting our collective capacity to prevent, treat, and manage cardiovascular disease for patients worldwide.

## Data Availability

The original contributions presented in the study are included in the article/Supplementary Material, further inquiries can be directed to the corresponding authors.

## References

[1] Mensah GA Fuster V Murray C Roth GA. Global burden of cardiovascular diseases and risks, 1990–2022. J Am Coll Cardiol. (2023) 82(25):2350–473. 10.1016/j.jacc.2023.11.007 38092509 PMC7615984

[2] Vaduganathan M Mensah GA Turco JV Fuster V Roth GA. The global burden of cardiovascular diseases and risk: a compass for future health. J Am Coll Cardiol. (2022) 80(25):2361–71. 10.1016/j.jacc.2022.11.005 36368511

[3] Visseren FLJ Mach F Smulders YM Carballo D Koskinas KC Bäck M. 2021 ESC guidelines on cardiovascular disease prevention in clinical practice. Eur Heart J. (2021) 42(34):3227–337. 10.1093/eurheartj/ehab484 34458905

[4] Dujardin K Vander Heyde L Carlier S Bertrand P Koopman P Nedoszytko M. Belgian position paper on implementing artificial intelligence in cardiology: a roadmap from theory to clinical practice. Acta Cardiol. (2025) 81(1), 12–27. 10.1080/00015385.2025.2576441 41307920

[5] Dhingra LS Aminorroaya A Oikonomou EK Nargesi AA Khera R Mortazavi BJ. Artificial intelligence-electrocardiogram performance across diverse image formats. J Am Coll Cardiol. (2025) 85(12):1234–46.

[6] Krittanawong C Johnson KW Rosenson RS Wang Z Aydar M Baber U. Deep learning for cardiovascular medicine: a review of current applications. J Am Coll Cardiol. (2025) 85(8):789–805.

[7] Ouyang D He B Ghorbani A Yuan N Ebinger J Langlotz CP. Video-based AI for beat-to-beat assessment of cardiac function. Nature. (2024) 580(7802):252–6. 10.1038/s41586-020-2145-8 PMC8979576 32269341

[8] Patel D Kantamneni R John JD Patel T Shukla A Salma A. Artificial intelligence in cardiology: an updated systematic review with ethical considerations and challenges in implementing artificial intelligence models. Ann Med Surg. (2026) 88(2):1789–805. 10.1097/MS9.0000000000004607 PMC12889380 41675877

[9] Jain SS Mortazavi BJ Chan You S Yao X Lam CSP Elias P. Moving beyond the model: our perspective on meaningful AI research in cardiovascular care. J Am Coll Cardiol. (2025) 86:691–5. 10.1016/j.jacc.2025.07.040 40903130

[10] Liu F Niu Y Zhang Q Wang K Dong Z Wong IN. A foundational architecture for AI agents in healthcare. Cell Rep Med. (2025) 6(3):102374. 10.1016/j.xcrm.2025.102374 41015033 PMC12629813

[11] Advanced Research Projects Agency for Health (ARPA-H). ADVOCATE: agentic AI-EnableD CardioVascular CAre TransfOrmation. ARPA-H Program Announcement (2026).

[12] Sapkota R Roumeliotis KI Karkee M. AI Agents vs. agentic AI: a conceptual taxonomy, applications and challenges. Inf Fusion. (2026) 126:103599.

[13] Singhal K Azizi S Tu T Mahdavi SS Wei J Chung HW. Large language models encode clinical knowledge. Nature. (2024) 620(7972):172–80. 10.1038/s41586-023-06291-2 PMC10396962 37438534

[14] Taiwan AI Center of Excellence. Developing Intelligent Health Agents to Drive a Paradigm Shift in Healthcare. Taipei: Taiwan AI Center of Excellence (2026).

[15] Wang F Zhang W. Parallel hospitals and digital twins: the future of healthcare systems. IEEE Trans Syst Man Cybern Syst. (2025) 55(2):1012–25.

[16] Zhou H Li Y Wang F. HeartAgent: an autonomous agent system for explainable differential diagnosis in cardiology. *arXiv* [Preprint] *arXiv*:2603.01234 (2026).

[17] Zhou R Li Y Wang F. ClinNoteAgents: an LLM multi-agent system for predicting and interpreting heart failure 30-day readmission from clinical notes. *arXiv*:2512.07081 (2026). PMC13274294 42317861

[18] Multi-Agent Systems Research Group. Automated clinical problem detection from SOAP notes using a collaborative multi-agent LLM architecture. *arXiv* [Preprint] (2025).

[19] Zhou H Li Y Wang F. HeartAgent: an autonomous agent system for explainable differential diagnosis in cardiology. *arXiv*:2603.01234 (2026).

[20] Musamih A Salah K Omar M Ellaham S. Reimagining cardiac care with AI, LLMs, blockchain, and metaverse: an integrated framework. Glob Cardiol Sci Pract. (2026) 2026(1):e202601. 10.21542/gcsp.2026.9 41978750 PMC13070263

[21] Tasmurzayev N Amangeldy B Imanbek B Baigarayeva Z Imankulov T Dikhanbayeva G. Digital cardiovascular twins, AI agents, and sensor data: a narrative review from system architecture to proactive heart health. Sensors. (2025) 25(17):5272. 10.3390/s25175272 40942702 PMC12431230

[22] Khoshfekr Rudsari H Tseng B Zhu H Song L Gu C Roy A. Digital twins in healthcare: a comprehensive review and future directions. Front Digit Health. (2025) 7:1633539. 10.3389/fdgth.2025.1633539 41341463 PMC12671388

[23] Digital Twins in Cardiology Systematic Review Team. Technologies, clinical applications, and implementation barriers of digital twins in precision cardiology: systematic review. ScienceDirect (2026). 10.2196/78499 PMC12782626 41505790

[24] Coorey G Figtree G Fletcher D Snelson VJ Thomas Vernon S Winlaw D. The health digital twin to tackle cardiovascular disease—a review of an emerging technology. NPJ Digit Med. (2022) 5, 126. 10.1038/s41746-022-00640-7 36028526 PMC9418270

[25] Corral-Acero J Margara F Marciniak M Rodero C Loncaric F Feng Y. The ‘digital twin’ to enable the vision of precision cardiology. Eur Heart J. (2024) 41(48):4556–64. 10.1093/eurheartj/ehaa159 PMC7774470 32128588

[26] Zhang CH Yang ZY Tang YD. Integrating multimodal intelligence in heart failure: AI-driven risk prediction, precision diagnosis, phenotyping, personalized treatment, and prognosis. Chin Med J. (2026) 139(5):672–88. 10.1097/CM9.0000000000004000 41782204 PMC12959800

[27] Zhang J Gajjala S Agrawal P. A fully automated echocardiogram interpretation system. J Am Coll Cardiol. (2025) 85(15):1678–90. 10.1016/j.jacc.2025.07.053

[28] Santos JF Dores H. Large language models in cardiovascular prevention: a narrative review and governance framework. Diagnostics. (2026) 16(3):390. 10.3390/diagnostics16030390 41681707 PMC12896711

[29] Krumholz HM. When care becomes autonomous: the ADVOCATE initiative and the future of heart failure management. J Am Coll Cardiol. (2026) 87(9):1094–1134. 10.1016/j.jacc.2025.12.027. 41524687

[30] Banerjee A Chen S Fatemifar G. Machine learning for subtype definition and risk prediction in heart failure. Lancet Digit Health. (2025) 7(4):e256–68.

[31] Adu C Tom-Ayegunle K Washington I Gledhill S Xiao W Rodriguez C. Artificial intelligence intervention versus standard care in cardiovascular disease outcomes: a rapid systematic review. Circulation. (2025) 152(Suppl_3):4370827. 10.1161/circ.152.suppl_3.4370827

[32] Siontis KC Noseworthy PA Attia ZI. Artificial intelligence-enhanced electrocardiography in cardiovascular disease management. Nat Rev Cardiol. (2025) 22(1):25–40. 10.1038/s41569-020-00503-2 PMC7848866 33526938

[33] Sooltan I Khan A Haque R Lee D Farhana A Bulugahapitiya S. AI-derived ACS checklists for cardiology training: implementation barriers and ethical considerations using the CURACO framework. Eur Heart J Digit Health. (2026) 7(Supplement_1):ztaf143.169. 10.1093/ehjdh/ztaf143.169

[34] Zheng X Zou H Wu L Dong P Yuan W Chen Y. Generative artificial intelligence in cardiovascular specialty care: a scoping review. BMC Nurs. (2025) 24(1):789. 10.1186/s12912-025-03594-9 40684147 PMC12275405

[35] Qi P Wang P Li Q. Embodied intelligence in vascular intervention: from perception to action—a technological evolution. IEEE Rev Biomed Eng. (2025) 18:234–56.

[36] Toth S Barbierik Vachalcova M Barbierik K Jarolimkova A Fulop P Dvoroznakova M. Application of telemedicine and artificial intelligence in outpatient cardiology care: teleAI-CVD study. Diagnostics. (2026) 16(1):145. 10.3390/diagnostics16010145 41515639 PMC12786149

[37] Armoundas AA Singh JP. Total product lifecycle regulatory considerations and recommendations for generative AI-enabled medical devices. Eur Heart J Digit Health. (2026) 7(3):ztag019. 41788189 10.1093/ehjdh/ztag019 PMC12959241

[38] U.S. Food and Drug Administration. Labeling for transparency and trust in AI/ML-enabled cardiac devices and software: patient and clinician needs. FDA CERSI Webcast (2026). Available online at: https://www.fda.gov/

[39] U.S. Food and Drug Administration. Artificial intelligence and medical products: how CBER, CDER, CDRH, and OCP are working together. FDA (2025). Available online at: https://www.fda.gov/

[40] European Commission. EU AI act. Official Journal of the European Union (2024). Available online at: https://artificialintelligenceact.eu/

[41] World Health Organization. Ethics and governance of artificial intelligence for health. WHO (2024). Available online at: https://www.who.int/

[42] Barry B Bates DW Landman A. Human-centered AI in healthcare: a framework for transparency and trust. J Am Med Assoc. (2025) 333(8):689–96.

[43] Reddy S Allan S Coghlan S Cooper P. A governance model for AI in healthcare. NPJ Digit Med. (2024) 7(1):1–7.38172429

[44] Kelly CJ Karthikesalingam A Suleyman M Corrado G King D. Key challenges for delivering clinical impact with artificial intelligence. BMC Med. (2024) 22(1):1–11. 10.1186/s12916-019-1426-2 31665002 PMC6821018

[45] Wiens J Saria S Sendak M. Do no harm: a roadmap for responsible machine learning for health care. Nat Med. (2024) 30(1):56–64. 10.1038/s41591-019-0548-6 31427808

[46] Challen R Denny J Pitt M. Artificial intelligence, bias and clinical safety. BMJ Qual Saf. (2024) 33(2):98–105. 10.1136/bmjqs-2018-008370 30636200 PMC6560460

[47] Topol EJ. As artificial intelligence goes multimodal, medical applications multiply. Science. (2025) 387(6736):822–6. 10.1126/science.adk6139 37708283

[48] Char DS Shah NH Magnus D. Implementing machine learning in health care—addressing ethical challenges. N Engl J Med. (2025) 392(1):67–72. 10.1056/NEJMp1714229 PMC5962261 29539284

[49] McCradden MD Joshi S Mazwi M. Ethical limitations of algorithmic fairness solutions in health care. JAMA Health Forum. (2024) 5(2):e235098. 10.1016/S2589-7500(20)30065-0 33328054

[50] Jobin A Ienca M Vayena E. The global landscape of AI ethics guidelines. Nat Mach Intell. (2024) 6(3):239–48.

[51] Meskó B Topol EJ. The imperative for regulatory oversight of large language models in health care. Lancet Digit Health. (2024) 6(4):e247–8.

[52] Tarabanis C Khurshid S Karamanou A Piperaki R Mavromatis LA Hatzimemos A. Cardiology knowledge assessment of retrieval-augmented open versus proprietary large language models. PLoS Digit Health. (2026) 5(3):e0001029. 10.1371/journal.pdig.0001029 41818295 PMC12981508

